# Supplementary material for: The Preliminary Analysis of Flavonoids in the Petals of Rhododendron delavayi, Rhododendron agastum and Rhododendron irroratum Infected with Neopestalotiopsis clavispora
Source: Int J Mol Sci. 2024 Sep 4;25(17):9605. doi: 10.3390/ijms25179605 (PMC11394826; doi:10.3390/ijms25179605)
Supplement: Supplementary file 1 [file ijms-25-09605-s001.zip › Supplementary Figure.pdf]

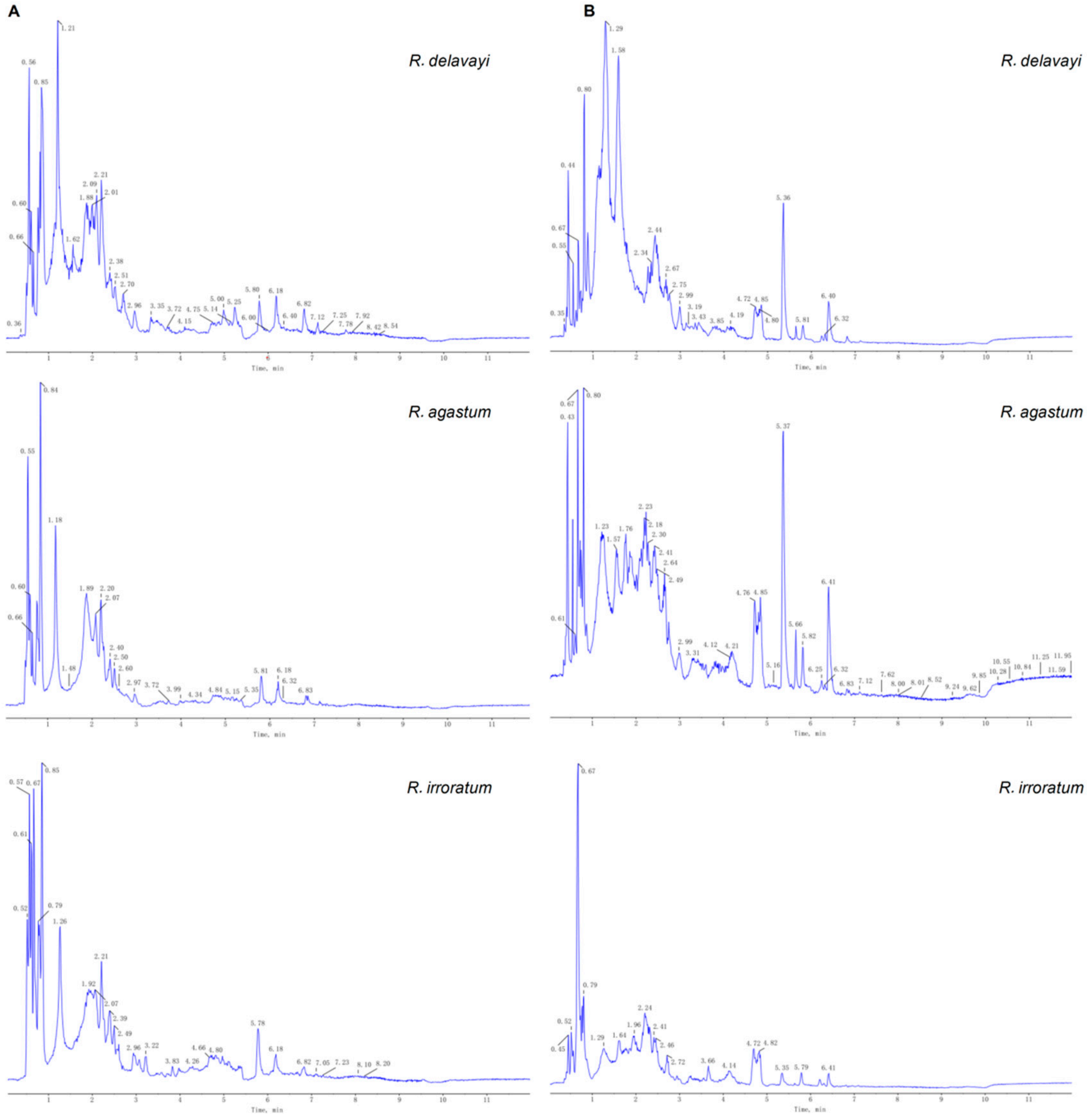

Supplementary Figure S1. Total ion current (TIC) chromatogram of *R. delavayi*, *R. agastum*, and *R. irroratum* petals. **(A)** TIC in positive mode. **(B)** TIC in negative mode.

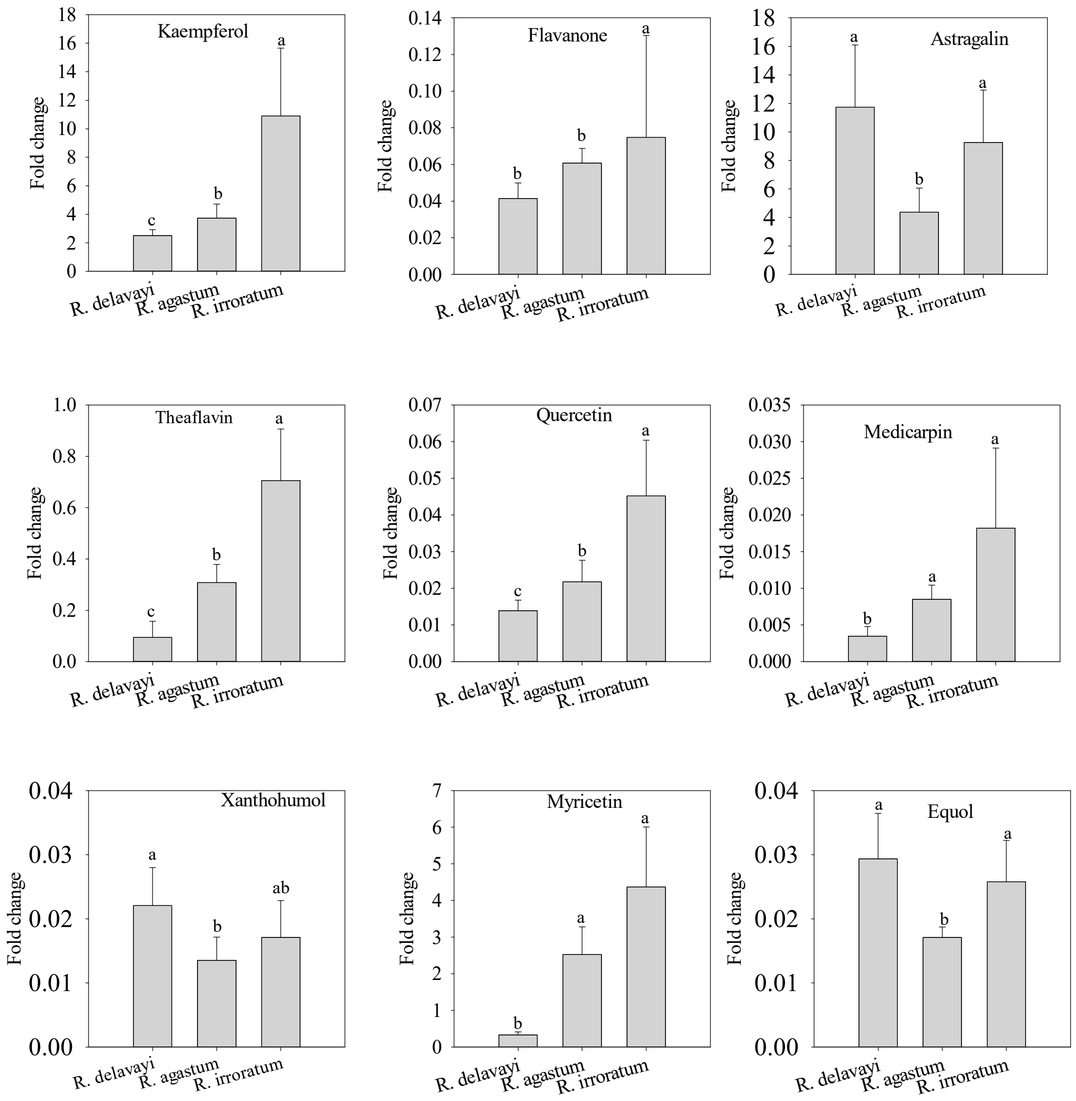

Supplementary Figure S2. The relative content of differential accumulated flavonoid in *R. delavayi, R. agastum, R. irroratum* petals. All metabolites are detected through the UHPLC-QTOF-MS in positive ion mode. The different lowercase letters on the bars indicate significant differences ($P < 0.05$).

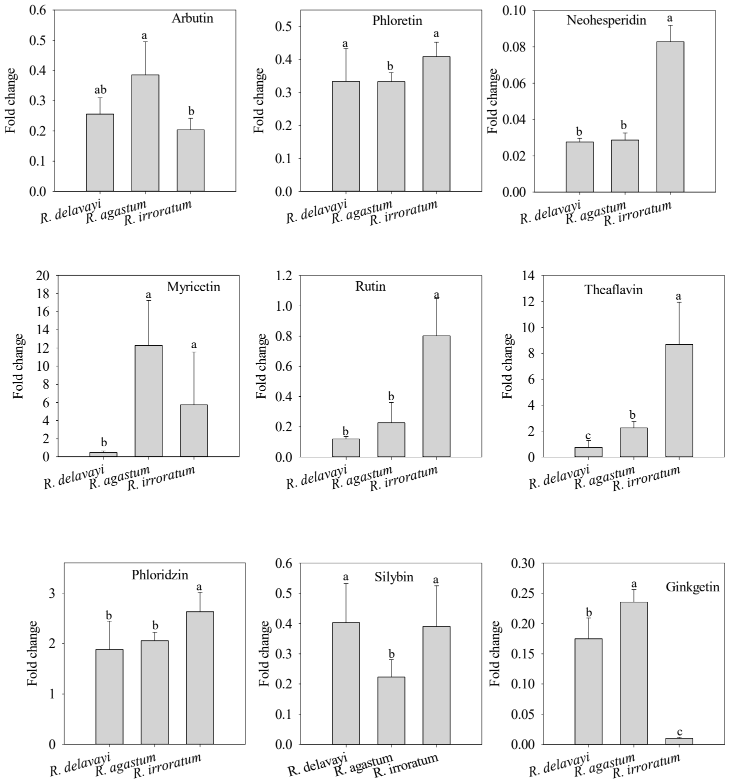

Supplementary Figure S3. The relative content of differential accumulated flavonoid metabolites in *R. delavayi*, *R. agastum*, *R. irroratum* petals. All metabolites are detected through the UHPLC-QTOF-MS in negative ion mode. The different lowercase letters on the bars indicate significant differences ($P < 0.05$).

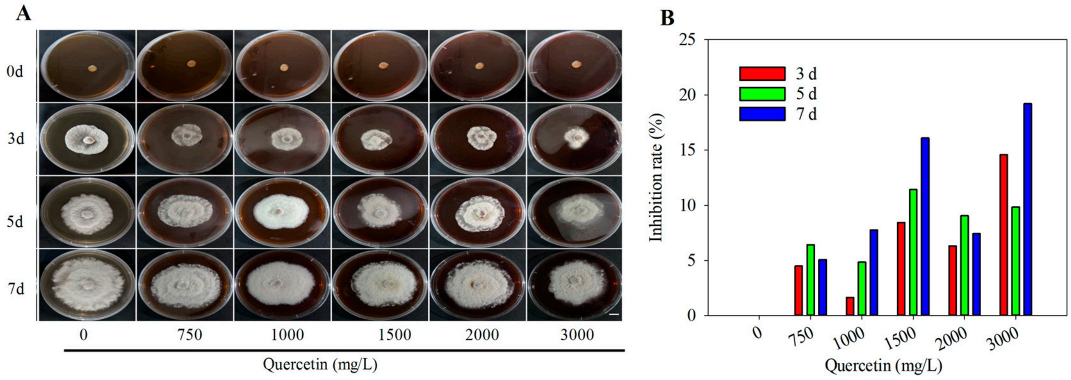

Supplementary Figure S4. The impact of quercetin on the mycelial growth of *N. clavispora*. (A) indicate the effects of varying concentrations of quercetin on the mycelial growth of *N. clavispora*. Bar = 1cm. (B), show the inhibitory rates of the mycelial growth of *N. clavispora* at different concentrations of quercetin.
